# Contrastive intonation effects on word recall for information-structural alternatives across the sexes

**DOI:** 10.3758/s13421-021-01174-1

**Published:** 2021-05-24

**Authors:** Xaver Koch, Katharina Spalek

**Affiliations:** Department of German Language and Linguistics, Humboldt-Universität zu Berlin, Unter den Linden 6, 10099 Berlin, Germany

**Keywords:** Focus, Alternatives, Memory, Delayed recall, Sex differences

## Abstract

Focus highlights the fact that contextual alternatives are relevant for the interpretation of an utterance. For example, if someone says: “The meeting is on TUESDAY,” with focus marked by a pitch accent on “Tuesday,” the speaker might want to correct the assumption that the meeting is on Monday (an alternative date). Intonation as one way to signal focus was manipulated in a delayed-recall paradigm. Recall of contextual alternatives was tested in a condition where a set of alternatives was evoked by contrastive intonation. A control condition used intonation contours reported for broad focus in German. It was hypothesized that contrastive intonation improves recall, just as focus-sensitive particles (e.g., ‘only’) do, compared to sentences without such particles. Participants listened to short texts introducing a list of three elements from taxonomic categories. One of the three elements was re-mentioned in a subsequent critical sentence, realized with either a broad (H+!H*) or with a contrastive intonation contour (L+H*). Cued recall of the list elements was tested block-wise. Results show that contrastive intonation enhances recall for focus alternatives. In addition, it was found that the observed recall benefit is predominantly driven by females. The results support the assumption that contextual alternatives are better encoded in memory irrespective of whether focus is expressed prosodically or by a focus-sensitive particle. The results further show that females are more sensitive to pragmatic information conveyed through prosody than males.

## Introduction

Focus denotes that part of an utterance that is important to a speaker or that the speaker considers to be most informative for the listener (e.g., Lambrecht, 1994). Both cross-linguistically and within a given language, there are different ways to signal focus: via prosody, morphology, or syntax. Common to all of these different types of marking is that they increase the prominence of a focused element within an utterance. This increased prominence, in turn, makes the focused element more salient to the listener. Increased salience has an effect on online language processing: Targets (phonemes, artificially added clicks, or entire words) are detected faster in focused than unfocused spoken language material (Cutler, 1976; Cutler & Fodor, 1979), focused phrases undergo deeper semantic processing (Wang et al., 2009; Wang et al., 2011), and focused phrases are retained better in memory (Birch & Garnsey, 1995; see review by Cutler et al., 1997, for more studies showing how prosodic prominence affects the integration of words in an existing discourse-model). However, it seems to be the case that there are notable individual differences in the perception and interpretation of prosodic prominence. Most notably, differences between the sexes have been reported (e.g., Wang et al., 2011).

The purpose of the present study was to further investigate the role of focus on memory, in particular on the recall of alternatives to the focused element that have been mentioned in the discourse context. A second research question concerns possible differences between men and women. In order to prepare the ground, we present the theoretical framework of Alternative Semantics (Rooth, 1985, 1992), discuss the difference between pragmatic and semantic uses of focus, review sex differences in pragmatic and prosodic processing, and summarize previous findings on focus and memory.

### Focus alternatives

Approaches to focus that make use of the notion of alternatives (most notably Alternative Semantics by Rooth, 1985, 1992, 2016, but also Roberts, 2012) assume that focus refers to “alternative phrasal meanings” (cf. Rooth, 2016: 19). We can distinguish between so-called pragmatic and semantic uses of focus (cf. Krifka, 2008; Velleman & Beaver, 2016). In pragmatic use, focus gives rise to a pragmatic inference. For instance, for “Mary introduced [BILL]_F_ to Sue” (example from Rooth, 1992), where the focused element is spoken with nuclear pitch accent (indicated here with capital letters), a listener might draw the inference that Mary introduced Bill to Sue but not to anybody else, negating alternative propositions like “Mary introduced [TOM]_F_ to Sue” or “Mary introduced [ANN]_F_ to Sue.” However, the inference can be cancelled, as in “Mary introduced [BILL]_F_ to Sue. And she introduced [ANN]_F_ to Sue.” By contrast, semantic use of focus affects the truth conditions of a sentence. This is most easily demonstrated for cases where a focus-sensitive operator like the particle “only” associates with the focus. The sentence “Mary only introduced [BILL]_F_ to Sue” asserts that of all the alternative individuals Mary could have introduced to Sue, she introduced Bill and nobody else to Sue. This assertion cannot be cancelled. If it turns out that Mary introduced a second person to Sue, then the first sentence states an untruth.

When we talk about alternative sets in this paper, we refer to the set of referents that can replace the focused referent in the given context. Following Rooth (1992), the focused element is also a member of the set. Thus, in the examples discussed above, the alternative set consists of {Bill, Tom, Ann}.

### Focus marking

In different languages, linguistic focus can be marked in different ways: Syntactically by employing non-canonical word orders like fronting or clefts, morphologically by adding focus markers, and prosodically by using particular intonation patterns. In German, as in English, prosodic focus marking is the most common. In German speech-production studies, Baumann, Becker, Grice, and Mücke (Baumann et al., 2007, see also Baumann et al., 2006) observed increases in prominence-lending parameters such as increased duration, higher pitch peak, an increased pitch excursion and hyperarticulation on the accented syllables in focused words. Different pitch accents can be identified based on pitch height and pitch excursion. Pierrehumbert and Hirschberg (1990) catalogued different accent types according to their function. L+H accents – that is, accents with a low starting point, are identified as conveying the salience of some scale. Out of this class of accents, Pierrehumbert and Hirschberg describe the L+H* accent as conveying “that the accented item – and not some alternative related item – should be mutually believed” (p. 296), that is, L+H* is used to mark contrastive focus. However, as Roessig, Mücke, and Grice (Roessig et al., 2019) point out, the mapping between focus type and pitch accent type is not quite as clear-cut as that: Grice et al. (2017) collected data from five participants producing the same words in broad, narrow, and contrastive focus conditions. The authors analyzed the realizations of accents both qualitatively and quantitatively. They found that not all speakers showed the expected categorical shift from H+!H* to H* to L+H* for broad, narrow, and contrastive focus, respectively. All speakers did show quantitative shifts of the three measured dimensions in the expected direction, though (see also Kügler & Gollrad, 2015, for similar results). As far as interpretation is concerned, Watson et al. (2008) showed (for English) that the interpretative domains of pitch accent types partly overlap: Participants listened to spoken instructions and had to manipulate objects presented on a display. Eye movements revealed participants’ anticipations of referents during on-line comprehension: While stimuli spoken with an L+H* accent strongly biased listeners’ anticipations to a contrastive referent, H* accents were compatible both with contrastive and new referents. Thus, there is a certain amount of variability both in the production and in the interpretation of pitch accents for focus.

In contrast to prosodic focus, which gives rise to a cancellable pragmatic inference and, as we have just seen, varies both in production and comprehension, so-called focus-sensitive particles like *only* or *also* are structure-sensitive operators that associate with a focused element and relate the value of the focused expression to a set of alternatives (Koenig, 1991: 33). Thus, reference to a set of focus alternatives is part of the meaning of a focus particle. A sentence containing an inclusive/additive focus particle presupposes that it is also true for at least one alternative proposition. A sentence containing an exclusive focus particle asserts that it is only true if it holds for no alternative proposition. Independent of the exact operation performed by the particle, it requires alternatives for its interpretation. This has been called “conventionalized sensitivity to focus” by Beaver and Clark (2008). We believe that the activation of alternatives in the presence of a focus particle might be “stronger” than after pitch accents because the particle serves as an additional, non-negligible cue for focus, whereas “mere” prosodic focus might be overheard or interpreted differently. As discussed in the previous paragraph, there is no one-to-one mapping between a given pitch accent type and its interpretative domain. Due to their clearly defined lexical meaning, focus particles resolve this ambiguity in favor of an interpretation requiring alternatives. In addition, in a sentence containing a focus particle, reference to alternatives is an integral part of the interpretation process which cannot be skipped.

### Memory representation of focused elements and alternatives

Fraundorf et al. (2010) contrasted two different accounts^1^ for the effect of prosodic focus on memory. The first account, the granularity account, has been proposed by Sturt, Sanford, Stewart, and Dawydiak (Sturt et al., 2004). These authors tested short-term memory effects of focus with a change-detection paradigm. Participants saw a set-up sentence and a critical sentence. Depending on the structure of the set-up sentence, the critical sentence was either in broad focus or had narrow focus on one of its constituents. The critical sentence was presented a second time, and participants had to indicate whether or not a word in the sentence had changed compared to the first presentation. Participants were better in detecting coarse violations as in replacing “the man with the hat” with “the man with the dog” compared to replacing it with “the man with the cap.” However, if “the man” was focused, participants detected more of these semantically close replacements. Sturt and colleagues therefore suggested that focus increases the granularity (the fineness) of the semantic analysis on a word, such that a word not in focus will be represented at a cruder granularity level than a word in focus (see also Sanford et al., 2006). More recent neurophysiological studies support the view that a focused element is subjected to a deeper, more fine-grained semantic analysis (Wang et al., 2009, 2011). A different account, dubbed “contrast account” by Fraundorf et al. (2010), assumes that the use of specific pitch accents not only modulates the representation of the accented word but also that of a contrast item or a set of contrast items, respectively. Fraundorf et al. (2010) found that contrastive pitch accents increased not only memory for a focused element but also memory for its alternatives. In particular, in their Experiment 3, they showed that a contrastive L+H* pitch accent very specifically improved participants’ ability to discriminate between the focused element for which a statement was true and a mentioned alternative (the “contrast item” in the authors’ terminology), for which the statement was false. In the following paragraphs, we present their study and two others (Fraundorf et al., 2013; Spalek et al., 2014) in some depth because these three studies form the basis of our own experiment.

Fraundorf et al. (2010) were the first to assess memory for focus alternatives. They presented participants with spoken little stories like example (1):

1. Both the British and the French biologists had been searching Malaysia and Indonesia for the endangered monkeys.
  Finally, the {British/ French} spotted one of the monkeys in {Malaysia/ Indonesia} and planted a radio tag on it.

The critical words ({British/ French}; {Malaysia/ Indonesia}) were either realized with an H* pitch accent or a (contrastive) L+H* pitch accent. Experiment 3 of Fraundorf and colleagues is most relevant for us: Participants had listened to the stories in the lab and returned a day later to answer recognition questions, for example:

(2) The British scientists spotted the endangered monkey and tagged it – true or false?

Participants’ accuracy in accepting a correct target was increased if it had previously been marked by an L+H* pitch accent compared to an H* pitch accent. That is, participants were more likely to respond “true” to (2) if they had heard “Finally, the BRITISH^L+H*^ spotted one of the monkeys …” the day before than if they had heard “Finally, the British^H*^ spotted one of the monkeys …”. Critically, contrastive prosodic focus also helped participants to reject a false claim. That is, they were more likely to respond “false” to (2) if they had heard “Finally, the FRENCH^L+H*^ …” previously, compared to “Finally, the French^H*^ …”. Focus marking did not affect performance for unrelated lures that had not been present in the alternative set presented on day one, like, for example, “Portuguese scientists.” That is, participants’ memory for mentioned alternatives increased if the focused item had been presented with an L+H* pitch accent, and they demonstrated this by remembering that the alternative had not been the element for which the proposition was true. In a later study, Fraundorf et al. (2013) repeated their findings with written stimuli where the focused element was realized with capital letters. In addition, they reported that the memory benefit only extends to contextually plausible alternatives.

Spalek et al. (2014) investigated the contribution of focus particles to the memory for focus alternatives: Participants listened to dialogues (Experiment 1) or narratives (Experiment 2), in which a set of three elements was introduced, see (3) and (4).

(3) Matthias receives a parcel with shirts, trousers, and jackets. He considered what he liked.
(4) He kept (a) only/ (b) even/ (c) the shirts.

After having listened to a block of ten of these little stories, participants had to answer questions about the dialogues. For critical items, the question was a recall question about the alternative set, for example: “What was in the parcel?” Spalek and colleagues looked at correctly recalled focus items (here: shirts) and alternatives (here: {trousers, jackets}) separately. While recall for the focus items was always better than recall for the alternatives, recall for the alternatives was improved in the presence of a focus particle compared to the control condition without a particle. There was no difference between *only* and *even*, that is, it did not matter whether the particle was inclusive or exclusive. Spalek and colleagues concluded that alternatives were made salient by a focus particle because reference to alternatives is part of the lexical meaning of a focus particle. This increased salience led to better memory encoding and hence, improved recall.

There are important differences between the studies by Fraundorf et al. (2010, 2013) and Spalek et al. (2014). Apart from the obvious language difference (Fraundorf et al.’s experiments were carried out in English and those of Spalek et al. in German), the alternative set introduced by Spalek and colleagues was larger (three elements) than the one introduced by Fraundorf and colleagues (two elements). Also, Spalek and colleagues used a recall task, whereas Fraundorf and colleagues investigated recognition memory. While recognition memory can rely at least to some extent on item familiarity, recall requires complete recollection of the studied episode (see Yonelinas, 2002, for a review on familiarity and recollection in memory). Most importantly, though, the manner of focus marking differed. Fraundorf and colleagues used prosodic focus marking, whereas Spalek and colleagues used focus-sensitive particles. As discussed above, the inference caused by prosodic focus marking is cancellable, that is, it can be taken back without causing any linguistics violations, and pitch accents like H* and L+H* do not map strictly onto information-structural categories like “new information” and “contrastive information.” Therefore, it seems likely that prosodic focus marking leaves more room for individual differences in processing. By contrast, reference to focus alternatives is a necessary part of the meaning of focus particles. That is, a focus particle “needs” alternatives in order to be interpreted, and hence, activating an alternative set is not optional. Seen like this, focus particles might be regarded as a stronger cue to the presence of alternatives than mere prosodic markings. One of the aims of the present experiment was to directly compare the study by Spalek et al. – including particles – with a replication where focus is marked with prosody only. Thus, the question is whether accent by itself can also make alternatives more salient and therefore improve their recall. Observing a memory benefit in recall for the focused element alone would support the granularity account, whereas observing a memory benefit either for recall of the focused element and its alternatives or for recall of the alternatives alone would join Fraundorf et al. (2010, 2013) and Spalek et al. (2014) as support for the contrast account.

In the present paper, we ask the following research questions: Do we observe a memory benefit for alternatives to prosodically focused elements in a recall task? That is, will we replicate the findings of Fraundorf et al. (2010) with a different task and with a larger alternative set (three items instead of two)? An additional question concerns the comparison of focus marked by prosody (arguably the weaker cue and the type of marking that allows for more individual variance) compared to focus marked by the additional presence of a focus particle. We use the same paradigm and the same stimuli as Spalek et al. (2014) (only slightly altered to replace focus marking with particles by focus marking through accent), which allows for a direct comparison of the results. Finally, we include participants’ sex as a factor in our design to test if females and males show equal memory benefit. In the following sections, we outline why we might expect differences between men and women.

### Sex differences in memory

It is well documented that women outperform men in episodic memory tasks if the material to be memorized is verbal, whereas men outperform women for episodic memory on visuo-spatial materials (e.g., Herlitz et al., 1997; Herlitz & Rehnman, 2008; Lewin et al., 2001).

In a meta-analysis on the brain areas underlying working memory, Hill et al. (2014) observed that women and men activate gender-specific networks during working memory tasks (see also Goldstein et al., 2005, for an fMRI study on auditory verbal working memory). While the neural differences were not accompanied by corresponding performance differences (in fact, groups were matched on working memory scores), they support the assumption that memories are encoded and retrieved somewhat differently between the sexes.

While the previously reported studies argue for a general memory benefit for women, women might also process prosodic cues differently from men and this might affect their memory for focus alternatives. In the next section we summarize findings supporting this hypothesis before turning to our study.

### Sex differences in semantic and intonation processing

It has been suggested that sex differences exist in the functional organization of the brain for language (e.g., Shaywitz et al., 1995). This finding has been hotly contested, with a number of reviews and meta-analyses unable to replicate these sex differences (e.g., Ihnen et al., 2009; Wallentin, 2009). Kaiser et al. (2009) find that sex effects in brain organization are very variable and depend on many factors, including choice of analysis and choice of threshold. This idea is seconded by Harrington and Farias (2008), who observed that, if the analysis method is held constant, sex effects do not obtain in all language tasks. Bearing this cautionary advice in mind, there is evidence that sex differences might underlie some areas of language processing that are relevant for the task used in the present paper:

Kansaku et al. (2000) observed sex differences in the distribution of neural activity in a story-listening task. They argue that sex differences only become apparent when subjects are required to process the global structure of sentences. Kaiser et al. (2007) found sex differences in lateralization (with stronger left lateralization for women) in a silent free narration task. Sex differences in the underlying brain activation were also obtained in a task testing coherent story comprehension and reasoning about false beliefs (Frank et al., 2015). These findings suggest that sex differences in language processing are particularly strong when participants have to process longer sequences of coherent text, which is exactly what participants had to do in the recall task used here. However, sex differences have also been obtained for processing single words. Wirth et al. (2007) report an earlier and longer-lasting N400 component in the EEG of women compared to men when participants had to passively read related or unrelated word pairs. The authors argue that women conducted a deeper semantic analysis on these word pairs than men (see also Wang et al., 2011).

Sex differences in the interpretation of intonation have most often been investigated in the context of emotional speech processing. Wildgruber et al. (2002) observed sex differences in the brain areas that were involved in a task where participants listened to two renderings of the same sentence, spoken by the same woman in a happy, neutral, sad, or angry voice, and they had to decide which of the two renderings was more “explicit.”

Findings from an EEG-study by Schirmer et al. (2002) on emotional speech processing suggest that female listeners integrate prosodic and semantic information much faster than male listeners. Schirmer et al. (2005) built on this finding by looking at whether men and women also differ in their pre-attentive reaction to neutral and emotional stimuli. They investigated the mismatch negativity in the EEG of participants who listened to bi-syllabic stimuli (da-da) spoken in neutral or emotional (angry/happy) prosody. While both sexes reacted to deviants by presenting the classic MMN component, only women varied in the size of the MMN depending on whether it was in response to an emotional or to a neutral deviant (see also Hung & Cheng, 2014, for very similar findings).

Furthermore, the results of a neuroimaging study by Schirmer et al. (2004) on emotional speech processing suggest that male listeners are less susceptible to intonation manipulations as evidenced by reduced IFG (inferior frontal gyrus) contrast activations for incongruent versus congruent speech presentation.

Given these findings in the literature, we a priori decided to test a gender-balanced sample in the present study to further explore the possible influence of participant sex on how prosodic focus is exploited when creating a representation of a just heard story.

## Study design and method

We set up a delayed recall experiment with auditory stimulus presentation and orthographic prompts. A combined within-subjects plus within-items design was employed with each participant and each experimental item tested in both experimental conditions: broad focus and contrastive focus.

### Participants

107 young adults were recruited from the participant database LingEx sustained by the Leibniz-Zentrum Allgemeine Sprachwissenschaft. All participants were native speakers of German, over 18 years of age, and 57 were female). Due to a clerical error, 13 subjects were tested although they had been tested shortly before using a similar stimulus set in a related fMRI experiment (Spalek & Oganian, 2019). These 13 participants (ten female) were excluded from the test sample. The remaining 94 participants (47 female) ranged in age from 18 to 39 years with an overall mean of 25.4 years (*SD* = 4.9; *M*^female^=25.4; M^male^=25.2).

None of the participants reported hearing loss, language disorders, or had a history of a neurological disease. The procedure was approved by the ethics committee of the Deutsche Gesellschaft für Sprachwissenschaft (German Linguistic Society, https://dgfs.de/de/inhalt/ueber/ethikkomission.html). All participants provided written informed consent and were instructed that they could withdraw from the study at any time.

### Materials

All materials are based on Experiment 2 in Spalek et al. (2014). Participants listened to short texts containing two context sentences and a critical sentence (cf. example (5)). The first context sentence introduced a set of three elements (nouns) from taxonomic categories (e.g., furniture, tools, fruits) and connected a person with the setting. The three elements (target words) from the first sentence ranged in frequency between 0.01 and 196 occurrences per million (cf. DLEX database; Heister et al., 2011). The norms for the taxonomic categories to which the target words belong can be found in Schröder et al. (2012). The second context sentence continued the story and, in most cases, indicated a choice or selection to be made by the person. The critical third sentence complemented the context sentences and re-mentioned one of the three elements from the first sentence. Half of the experimental items contained the definite determiner before the re-mentioned noun and half of them did not, depending on what sounded natural in the given contexts. Care was taken that, across items, the focused element in the critical sentence was equally often the first, second, or third element from the first context sentence. Two versions were constructed for each critical sentence: (a) a control condition with broad focus (H+!H* pitch accent) and (b) a version with a contrastive focus (L+H*pitch accent) on the aforementioned element.
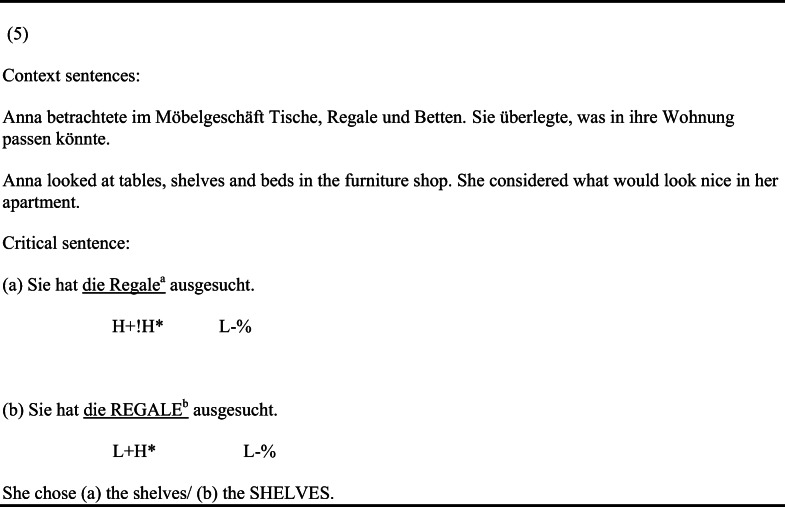


We used a set of 80 experimental items (45 targets and 35 fillers) plus six practice items. The fillers had the same structure as the targets and presented lists of three elements from various categories, either taxonomic or non-taxonomic (cf. Table 2 for examples). Half of the fillers were presented with broad focus and half with contrastive focus in the critical third sentence (see Appendix A for a transcription of all target items. All sound files are provided in the Online Supplementary Materials, https://osf.io/txq5r).

A professional female speaker was recorded in a sound-shielded booth using a directional Sennheiser (ME64) microphone and an Edirol E09 solid state recorder (44.1 kHz sampling frequency, 16-bit resolution). The speaker had a middle German accent close to the standard variety of German. Two versions of each critical practice, filler and target sentence were recorded – a broad focus version (H+!H* pitch accent on the critical word) and a contrastive focus version (L+H* pitch accent on the critical word). Note that, in contrast to the stimuli used in Spalek et al. (Spalek et al., 2014, Experiment 2), no focus-sensitive particles (e.g., “only”) were used in the present study. The difference in the information structure for the critical sentences resulted from *prosodic* focus and was thus signaled by means of *intonation*.

The critical sentences for the *broad* focus condition were elicited with the pre-recorded wh-question “Was war los?” (“What happened?”) produced with a typical declining intonation contour (H* L-%; GToBI notation, cf. Grice & Baumann, 2002). The resulting *broad* focus answers of our speaker showed the German default declarative accentuation pattern, which assigns an H+!H* tone accent (GToBI notation) to the re-mentioned noun (L-% sentence final edge tone, GToBI). *Contrastive* focus productions of the critical sentences were responses to pre-recorded yes/no questions like “Sie hat die TISCHE ausgesucht?” (“She chose the TABLES?”). These yes-no questions were realized with a low nuclear tone accent (L*; GToBI) associated with the target noun (TABLES in the above example) followed by a high-rising interrogative contour (H-^H% edge tone, GToBI). Our speaker’s *contrastive* focus answers were characterized by a low-rising nuclear tone accent (L+H*, GToBI) on the focused constituent (i.e., the re-mentioned noun) in the critical third sentences and showed a default low sentence final edge tone (L-%, GToBI).

**Fig. 1:**
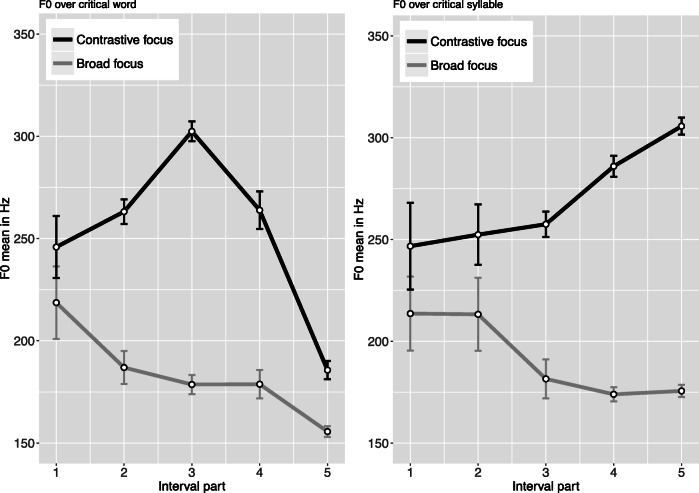
Mean pitch contour of focused element in the critical sentences for contrastive and broad focus condition (**left:** F0 contour across critical word; **right:** F0 contour for critical syllable; target items only). Error bars represent the standard error of the mean

**Fig. 2:**
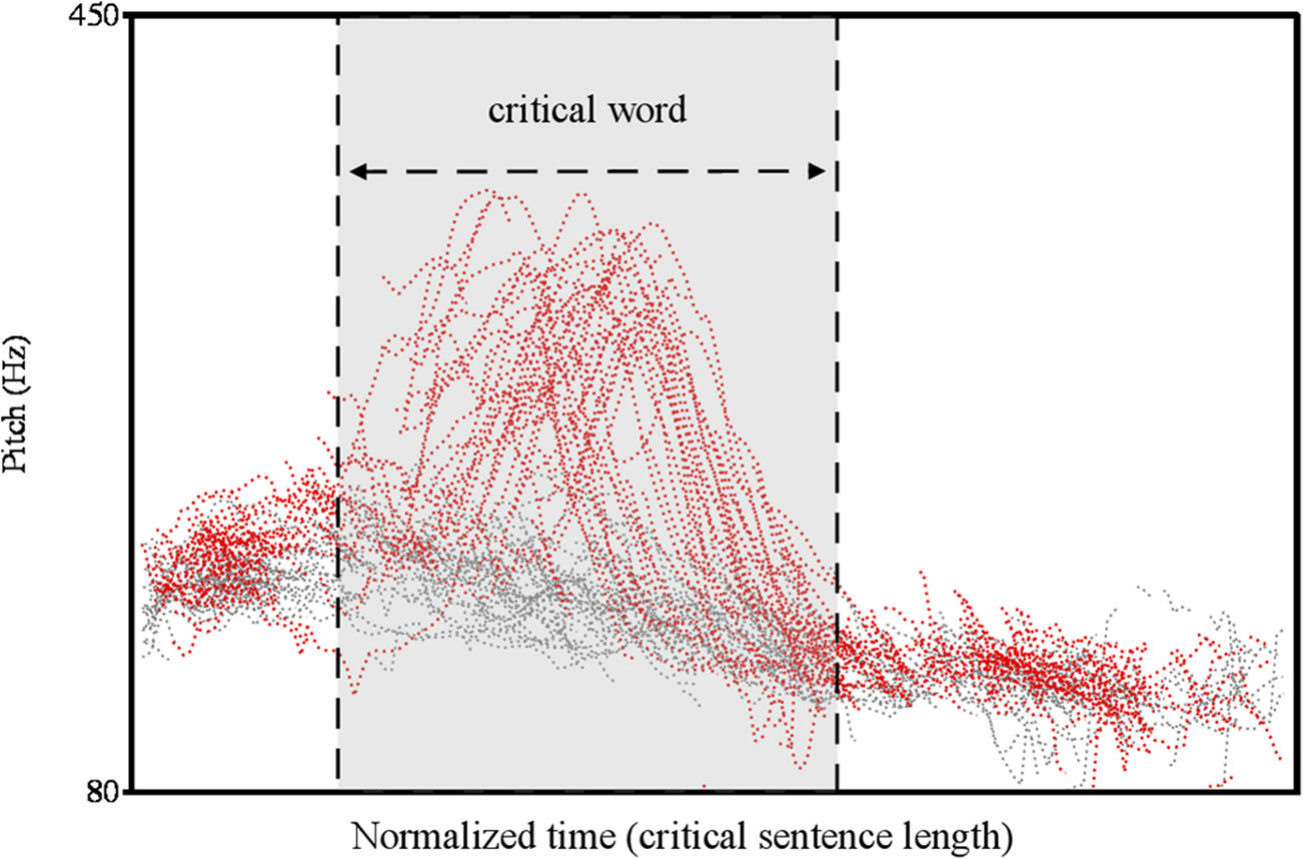
Pitch contours of the critical sentences (n=90) for contrastive vs. broad focus condition (gray: broad focus pitch contours; red: contrastive focus pitch contours); dashed window roughly indicates the position of the critical word

Acoustic analyses conducted with Praat (Boersma & Weenink, 2018, Version 6.0.16) confirmed that the critical elements were longer, had an increased sound pressure level and showed a steeper F0-excursion for the contrastive condition (L+H* pitch accent) compared to the broad focus condition (H+!H* pitch accent). Table 1 presents means and standard errors for the acoustic parameters duration, mean intensity, maximum and minimum pitch as well as pitch range for both focus conditions. Figure 1 displays averaged intonation contours of the re-mentioned elements (focused words) and the respective lexically accented syllable for the two focus conditions. Figure 2 illustrates the pitch contours for the duration of the critical sentences for both focus conditions.

**Table 1:** Mean acoustic parameters of the re-mentioned elements in the critical sentences (e.g., *Regale* in (5a, 5b), target items only)

*Analysis interval*/Measure	Contrastive focus		Broad focus			
	Mean	SEM	Mean	SEM	F(1,44)	*p*
*Critical word*						
Duration (ms)	541	11	496	10	36.95	<.001
Maximum pitch (Hz)	323	4	202	5	694.88	<.001
Minimum pitch (Hz)	157	2	145	1	12.45	.001
Pitch range (Hz)	165	5	57	5	397.23	<.001
Mean Intensity (dB)	74.10	0.30	70.78	0.27	83.89	<.001
*Stress syllable*						
Duration (ms)	294	7	272	7	19.14	<.001
Maximum pitch (Hz)	316	3	191	2	971.44	<.001
Minimum pitch (Hz)	233	4	166	2	149.55	<.001
Pitch range (Hz)	83	5	25	1	78.32	<.001
Mean Intensity (dB)	75.16	0.29	71.25	0.32	83.61	<.001

The 45 target items were presented on a pseudo-randomized list, interspersed with the 35 filler items. Two versions of this randomization list were employed (A and B). These two versions complemented each other in terms of focus condition for the target items. That is, a target item with broad focus on version A of the list was presented with contrastive focus on version B. The sequence of the items (targets and fillers) was identical for both versions of the presentation list. All in all, the experiment contained eight experimental blocks (ten items each, six or seven targets per block) plus a unique initial training block with six items (four filler-like items, two target-like items). Stimuli were randomized within blocks such that neither the same stimulus type (target, filler) nor the same focus condition (broad, contrastive focus) was presented more than three times in a row. In addition, taxonomic categories did not reoccur within blocks. Moreover, stimuli with identical taxonomic categories were arranged in experimental blocks with maximal distance from each other. Participants were quasi-randomly assigned to one of the two versions of the presentation list (participants’ sex was balanced across lists). That is, each participant heard a target item only once in a particular focus condition. Twenty-three out of 45 targets were presented with contrastive focus on version A of the list (22 targets realized with broad focus) and vice versa on version B. Seventeen out of 35 fillers were presented with contrastive focus and 18 with an intonation contour for broad focus on both versions of the list.

### Apparatus

Participants were seated in front of a Belinea TFT monitor and wore a Sennheiser PC131 headset with an integrated microphone. Stimulus presentation and recording of the answers was controlled by Neurobehavioral Systems Presentation software (Version 16.5).

### Procedure

The experiment started with an on-screen instruction informing participants about the structure of the experiment and the task they would have to perform. The experiment consisted of two paired phases: (a) an encoding phase (ENC) during which the participants listened to the short texts and (b) a cued recall phase (REC). Thus, phases of auditory presentation alternated with test phases in which participants were cued for overt recall. Subjects were informed that the recall phase was time-limited and that they were supposed to respond aloud. After the instructions, subjects performed six practice trials (a block of six three-sentence texts (ENC) followed by six questions (REC)) and were allowed to adjust the sound volume of the headphones. Table 2 presents a schematic of the experiments’ procedure.

**Table 2:** Experimental procedure

Example experimental block			
Encoding Phase	Trial type	German transcript	English transcript
Presentation: 1	Target	Anna betrachtete im Möbelgeschäft Tische, Regale und Betten. Sie überlegte, was in ihre Wohnung passen könnte. Sie hat Regale/**REGALE** ausgesucht.	Anna looked at tables, shelves, and beds in the furniture shop. She considered what would look nice in her apartment. She chose shelves/**SHELVES**.
Presentation: 2	Target	Robert suchte in seiner Werkstatt Zangen, Hämmer und Schrauben. Er schaute sich eine Weile um. Er hat die Zangen/**ZANGEN** gefunden.	Robert searched for pliers, hammers, and screws in his garage. He searched for a while. He found the pliers/**PLIERS.**
Presentation: 3	Filler	Susanne reinigte im Waschsalon Vorhänge, Tagesdecken und Fuβmatten. Sie benutzte auβerdem den Trockner. Sie hat die Tagesdecken getrocknet.	Susanne cleaned curtains, bedspreads, and door mats at the laundromat. She also used the dryer. She dried the bedspreads.
Presentation: 4	Target	Carsten griff in einen Korb mit Pfirsichen, Kirschen und Bananen. Er überlegte sich, worauf er Appetit hatte. Er hat die Pfirsiche/**PFIRSICHE** herausgeholt.	Carsten reached for a basket full of peaches, cherries, and bananas. He wondered what he would like to eat. He took out the peaches/**PEACHES.**
Presentation: 5	Filler	Renate überprüfte im Flugzeug Sitze, Gepäckfächer und Anschnallgurte. Sie entdeckte einige Defekte. Sie hat die Sitze beanstandet.	Renate checked seats, overhead lockers, and safety belts in the plane. She discovered some defects. She complained about the seats.
Presentation: 6	Target	Saskia traf auf dem Wochenmarkt Bäcker, Gärtner und Bauern. Sie wollte selbst einen Stand aufmachen. Sie hat sich mit den Bauern/**BAUERN** abgesprochen.	Saskia met bakers, gardeners, and farmers at the market She wanted to have her own market stall. She talked to the farmers/**FARMERS.**
Presentation: 7	Filler	Franz verkaufte in seiner Apotheke Augentropfen, Wärmepflaster und Nasenspray. Er überprüfte den Vorrat. Er hat Wärmepflaster nachbestellt.	Franz sold eyedrops, hot patches, and nasal spray in his pharmacy. He checked the supplies. He reordered hot patches.
Presentation: 8	Target	Janine betrachtete in der Ausstellung Statuen, Gemälde und Fotografien. Sie sollte eine Rezension schreiben. Sie hat die Gemälde/**GEMÄLDE** erwähnt.	Janine looked at statues, paintings, and photographs at the exhibition. She had to write a review. She mentioned the paintings/**PAINTINGS**.
Presentation: 9	Filler	Maja hatte ein Bilderbuch mit Adlern, Pelikanen und Papageien. Sie kannte schon einige Vögel. Sie hat die **PAPAGEIEN** erkannt.	Maja had a picture book with eagles, pelicans, and parrots. She already knew a number of birds. She recognized the parrots/**PARROTS**.
Presentation: 10	Target	Petra legte auf ihren Schreibtisch Füller, Blöcke und Locher. Sie musste etwas vorbereiten. Sie hat Füller/**FÜLLER** benutzt.	Petra put fountain pens, notepads, and hole punchers on her desk. She had to prepare something. She used fountain pens/**FOUNTAIN PENS**.
Recall Phase			
Cue/Question: 1	Target	Welche Möbel gab es im Möbelgeschäft?	Which pieces of furniture were there in the furniture shop? Expected response: tables, shelves, beds
Cue/Question: 2	Target	Welche Werkzeuge wurden in der Werkstatt gesucht?	Which tools had been searched for in the workshop? Expected response: pliers, hammers, screws
Cue/Question: 3	Filler	Was hat Susanne mit den Tagesdecken gemacht?	What did Susanne do with the bedspreads? Expected response: She dried them.
Cue/Question: 4	Target	Welches Obst lag zunächst im Korb?	Which fruits were there in the basket at first? Expected response: peaches, cherries, bananas
Cue/Question: 5	Filler	Was hat Renate im Flugzeug gemacht?	What did Renate do in the plane? Expected response: checked plane, complained about seats
Cue/Question: 6	Target	Welche Personen waren auf dem Wochenmarkt?	Which persons were there at the market? Expected response: bakers, gardeners, farmers
Cue/Question: 7	Filler	War die Person in der Apotheke ein Mann oder eine Frau?	Was the pharmacist male or female? Expected response: male
Cue/Question: 8	Target	Welche Kunstwerke gab es in der Ausstellung?	Which art objects were there at the exhibition? Expected response: statues, paintings, photographs
Cue/Question: 9	Filler	Was für ein Buch hat sich Maja angeschaut?	What kind of book did Maja look at? Expected response: picture book
Cue/Question: 10	Target	Welche Büromaterialien waren auf dem Schreibtisch?	Which office supplies were there at the desk? Expected response: pens, notepads, hole punchers
		10-step backwards counting task (e.g. 8-decrement)	80-72-64-56-48-40-32-24-16-8-0
**subsequent** **experimental block**			
Encoding Phase Recall Phase Backwards counting task			
[…] **Block 8**			

In the encoding phase, each trial began with a central fixation cross displayed for 500 ms. Then, the three-sentence text was presented auditorily, sentence by sentence with a silent pause of 500 ms duration between sentences. After a silent interval of 4 s the next trial started automatically. The average trial length in the encoding phase was about 12 s. Once ten texts (i.e., one ENC block) had been presented, the recall phase was initiated automatically. First, a screen informed the participants that the phase with the queries was about to begin (display duration 2 s.). Then, after a central fixation cross was presented for 500 ms, a test cue (question) appeared on the screen for 3 s followed by a ‘#’ symbol that was displayed for the remaining trial duration. Subjects were supposed to respond aloud as soon as the ‘#’ symbol appeared on the screen. Two types of questions were used as recall cues depending on whether the respective trial was a filler or a target. For the target items the questions aimed at the three elements presented in the first sentence of the text (e.g., “Which pieces of furniture were there in the furniture shop?” for example (5), above). For filler trials we asked 25 information questions and 10 yes/no questions on the texts presented in the respective encoding phase. These questions aimed at the action performed (e.g., “What did Susanne do with the bedspreads?”), the name or sex of the person involved (e.g., “What was the name of the person who ate ice cream?”), the location of the action (e.g., “Where was Sophie?”) or the element that was re-mentioned (e.g., “What was it that Stefan lost during the rugby game?”). The set of fillers was included to discourage participants from memorizing the list of three elements only. Participants had 20 s to respond aloud and their answers were recorded. In order to not lose information if a participant responded too early, that is, while the question was still on the screen, recordings actually started from the onset of the test cue. Participants could terminate trials with a button press as soon as they thought their answer was sufficient. We instructed the participants to indicate aloud if they did not know the answer to a question and *then* to press the button to terminate the respective trial. Immediately after the time out or after the participants’ button press, the next trial was initiated. All items were tested for recall in the same order as presented during the encoding phase. Thus, the number of trials and approximately the amount of time delay between encoding and recall was kept constant and subjects could easily keep track of the sequences. Between blocks, participants were asked to perform a ten-step n-backward counting task using decrements of two to nine and to take a small self-paced break. This procedure should reduce interference effects between blocks, because some taxonomic categories (e.g., fruit, vegetables, animals, furniture, plants) were used more than once in the experiment (but only once within a given block). After the experiment, a questionnaire was administered asking the participants for basic demographic information, what they thought the experiment was about, and whether they employed any specific strategies. An entire testing session lasted about 50 min. The participants were paid €8 in compensation.

## Results

The recorded answers were transcribed and recall accuracy per word was coded independently by an assistant annotator and the first author. Recall for each word was coded binarily, that is, a word was coded as “recalled“ (1), “not recalled“ (0), or it was excluded (NA). If participants mentioned a synonym (e.g., “shovel” for “spade”; cf. Duden, 2007, 2014, 2018) or a hyponym instead of a target word (e.g., “handbag” instead of “bag”) we coded this as a correct response. Cases of coding mismatch between the two raters were discussed with the second author and a binding coding scheme was established. Appendix B presents all synonym and hyponym decisions used in our coding scheme. If a participant indicated that he/she did not know an answer for a whole trial this was coded as not recalled (0) for all three words to be recalled for the respective item. One experimental item (item 44) was excluded from the analyses, because it resulted in uncertainty about the correct answer in the recall phase for a majority of participants. Further, 34 trials (out of 4,136 trials, i.e., approx. 0.8% of the data) could not be analyzed because the respective audio recordings were empty. We decided to exclude these trials completely, because we could not decide afterwards whether the data loss was due to technical failure or because participants forgot to indicate that they did not know an answer to a question. The raw data are available in the Online Supplementary Materials (https://osf.io/txq5r).

**Fig. 3:**
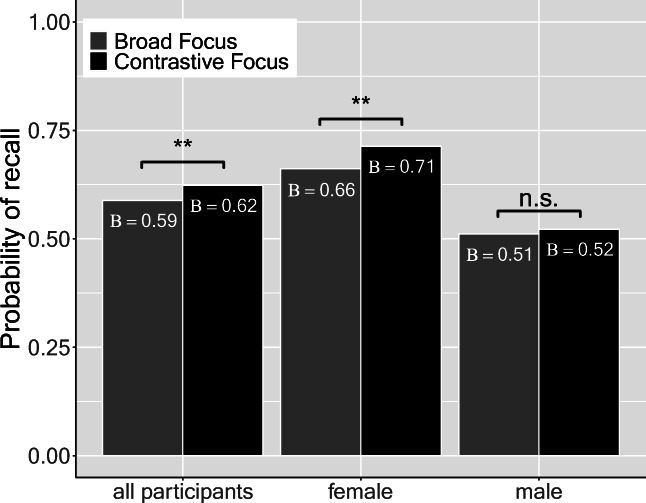
Model predictions for recall probability of contextual alternatives. Bar charts split by focus. **Left:** Pooled results. **Middle:** Female data. **Right:** Male data

Since we were primarily interested in the recall of the alternative set, we carried out two analyses, splitting the data into recall of the alternatives (e.g., “tables” and “beds” in example (5)) and into recall of the focused element (e.g., “shelves” in (5)). First, we analyzed the effect of contrastive focus on the probability to recall contextual alternatives (from here onwards: recall probability) to the focused element. Descriptive statistics pointed towards higher recall probability in the contrastive focus condition (*M* = 57.6% contextual alternatives correctly recalled) compared to the broad focus baseline condition (*M* = 55.1% contextual alternatives correctly recalled). Moreover, recall accuracy (across both focus conditions) was considerably lower for male than for female participants. We applied logistic mixed models in the statistical computing environment R (version 4.0.0) with the lme4 package (version 1.1-23) (cf. Bates et al., 2015) to assess the effects of focus and participants’ sex as well as their interaction on recall for contextual alternatives. Focus condition (broad focus vs. contrastive focus) and sex (male vs. female) were sum-coded. We started out with the maximal random effects structure given the experimental design, as advocated in Barr, Levy, Scheepers, and Tily (2013) (formula: glmer(recall~focus*sex+(1+sex|item)+(1+focus|participant)+(1|word),family=binomial, control=glmerControl(optimizer = "bobyqa",optCtrl=list(maxfun=2e5)))). “Item” refers to a story (e.g., example 5), “word” to a single word (e.g., “tables” in example 5). Random slopes were added to allow for the possibility that the effect of intonation differed across participants and that there were sex-dependent random effects of item. Likelihood-ratio tests (with the “anova()”-function) showed that the random slope for intonation improved model fit only marginally (*χ*^2^(2) = 5.15, *p* = .076), and it was therefore left out. Four control variables were tested via likelihood-ratio tests for their potential effects on recall for contextual alternatives: the version of the randomization list, position of the focused word in the list of elements (scaled and centered), target word frequency,^2^ and (scaled and centered) trial number (*χ*^2^(1) = 6.39, *p* < .05). Allowing for the possibility that the effect of trial number differed across participants by adding a random slope for trial number on the participant intercept drastically improved model fit (*χ*^2^(2) = 63.7, *p* < .001). Finally, we tested (based on the suggestion of an anonymous reviewer) whether adding the quadratic component of trial number by using a polynomic predictor further improved model fit, which it did (*χ*^2^(4) = 14, *p* < .01). The resulting parsimonious model (cf. Table 3) showed facilitatory effects of contrastive focus (*B* = 0.15, |*z|* = 2.83, *p* = .005) and trial number on recall probability for contextual alternatives (*B* = 25.83, |*z|* = 3.09, *p* = .005, linear component). Contrastive intonation on the focused element improved recall probability for contextual alternatives by 3.5% compared to the broad focus condition (cf. Fig. 3, left bar plot). Recall probability was higher for trials later in the experiment compared to earlier trials. In accordance with the descriptive findings above, the model revealed that male participants showed a generally lower recall probability for alternatives (-18.6%) as evidenced by a sex effect on contextual alternative recall (*B* = -0.79, |*z|* = 3.24, *p* = .001). Furthermore, we found a focus × sex interaction (*B* = -0.20, |*z|* = 1.97, *p* = .049), that is, a *reduced* focus effect on recall probability for contextual alternatives in male compared to female participants.

**Table 3:** Fixed-effect estimates (top) and variance estimates (bottom) for GLMER of recall for contextual alternatives (recall probability ~ focus * sex + poly(trial number,2) + (1+sex | item) + (1| word) + (1+poly(trial number,2) | participant), *n* = 8271, log-likelihood: -4705), coding scheme: sum coding

Fixed effects	Coefficient (*B*)	*SE*	*|z|*	P
Intercept	0.43	0.16	2.77	
Intonation focus	0.15	0.05	2.83	.005
Sex	-0.79	0.24	3.24	.001
Poly (Trial number(centered),2) 1	25.83	8.37	3.09	.002
Poly (Trial number(centered),2) 2	-7.53	8.39	0.90	.370
Intonation focus * Sex	-0.20	0.10	1.97	.049
**Random effects**	Variance			
Participant (Intercept)	1.30			
Random slope: poly(Trial number,2)1	896.12			
Random slope: poly(Trial number,2)2	320.58			
Item (Intercept)	0.33			
Random slope: Sex	0.11			
Word (Intercept)	0.18

Given that the interaction between focus and sex was significant, we carried out separate analyses for females and males. The models were identical to the full models (apart from the fact that the main effect of sex and the random slope of sex on the item intercept were no longer included). For females, the effect of focus was significant (*B* = 0.24, |*z|* = 3.24, *p* = .001), as was the linear component of (centered) trial number (*B* = 17.82, |*z|* = 2.34, *p* = .020). For males, the only significant effect was an effect for the linear component of (centered) trial number (*B* = 18.31, |*z|* = 2.78, *p* = .005). Critically, the effect of intonation was not significant (*B* = 0.04, |*z|* = 0.62, *p* = .537)

Second, we analyzed the effect of prosodic focus on the recall probability of the focused element. Descriptive statistics showed that the focused element was correctly recalled in 68% of the trials in the broad focus condition and in 69% of the trials in the contrastive focus condition. We started out in the same way as for alternative recall, with the maximal model justified given the design. Since there was only one focused word per item, we did not need separate random effects for word and item but used only one. We eliminated random slopes if they did not improve model fit. Again, control predictors were added to the most parsimonious model one by one and kept if they improved model fit. The final model contained fixed effects of focus condition and sex and their interaction and a fixed effect for experimental list. We modelled random intercepts for participants and items and the random slope for focus condition on the participant intercept. This model revealed that the recall of the focused element was not affected by focus (*B* = 0.06*, |z|* = 0.65, *p* = 0.51), but again by participants’ sex (*B* = -0.84*, |z|* = 4.07, *p* < .001). A summary of this model and a figure of the model predictions is presented in Appendix C.

**Fig. 4:**
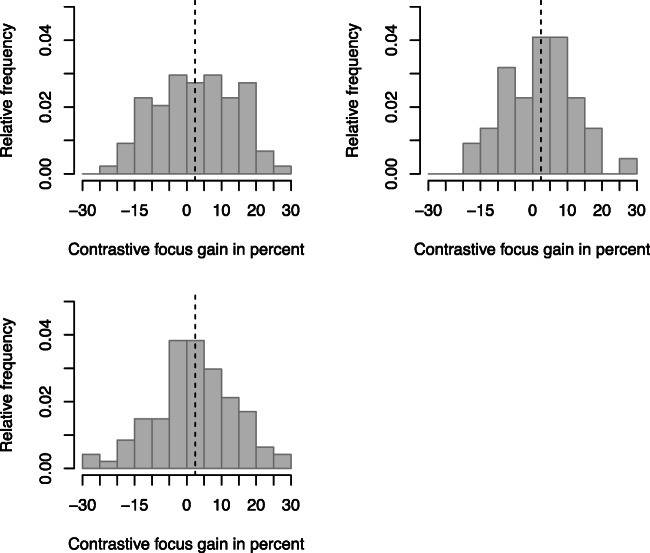
Histograms for contrastive focus gain (100*(contrastive recall – broad recall)) across three aggregated datasets. **Upper left:** aggregated across target words. **Upper right:** aggregated across items. **Lower:** aggregated across participants

**Fig. 5:**
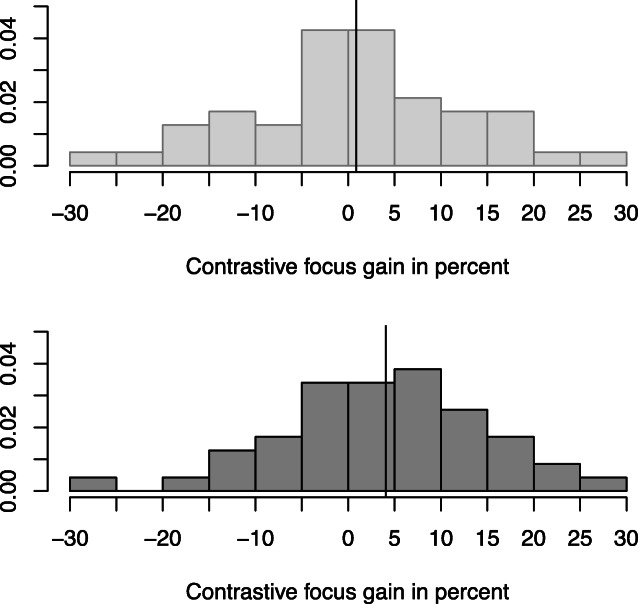
Aggregated across participants per sex; means displayed as solid lines in histograms. **Upper:** male participants. **Lower:** female participants

In order to explore whether the focus × sex interaction for the recall of contextual alternatives described above may be associated with distributional differences between male and female participants (beyond a shift in mean), we analyzed the variability of the contrastive intonation effects on recall probability (for alternatives). We summarized the effect of intonation on recall probability in a single variable per participant: *gain*. Gain corresponds to the percent contextual alternatives recalled if the focused element is presented with contrastive focus minus percent contextual alternatives recalled if the focused element is realized as broad focus. Influential subgroups with differential susceptibility regarding the prosodic manipulation may result in a non-normal (e.g., bimodal) distribution for contrastive focus *gain*. Such a finding would call for additional studies testing a battery of covariates that may account for such susceptibility differences (e.g., pitch discrimination abilities, ability to spot contrastive intonation). Moreover, participants may exist, who, while they are able to discriminate contrastive from non-contrastive intonation, do not benefit from contrastive intonation. In this case we should also observe deviations from the normal distribution across participant data or across data subsets split by participants’ sex. Consequently, we calculated the contrastive focus gain for all observational units separately in order to be able to visualize the order of magnitude of the contrastive focus gain and its dispersion. Figure 4 shows the distributions of contrastive focus gain aggregated over target words, items, and participants. Target-, item-, and participant-related distributions more or less resemble each other: the distributions did not violate the assumptions of a standard normal distribution (Shapiro-Wilk Test, *p* > .05 for the three datasets), and they were unimodal with a mean of approximately 2–3% contrastive focus gain. Figure 5 illustrates that female participants show more contrastive focus gain compared to male. However, both male and female histograms look similar except for the fact that the female distribution is skewed towards higher values. Neither of the distributions violates the assumptions of the standard normal distribution (Shapiro-Wilk Test, *p* > .05 for both datasets).

**Fig. 6:**
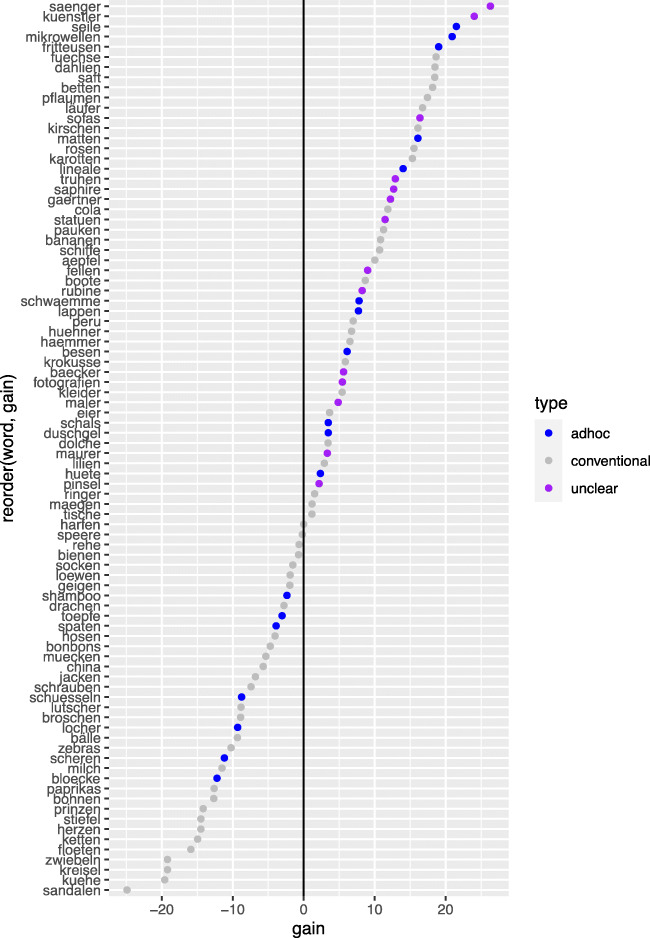
Contrastive focus gain for each word. Positive values indicate improved alternative recall if the focused element was produced with a contrastive accent

Based on the suggestion of an anonymous reviewer, we also did a post hoc investigation of the item variability: While all words were introduced as hyponyms to a hyperonym that was named in the third critical sentence, some of these hyperonyms were conventional, like, for example, FURNITURE or FRUIT, whereas others were more ad hoc like CLEANING UTENSILS or WRITING MATERIALS. We classified the items as “conventional” (n = 28) and “ad hoc” (n = 9). A further seven items were classified as “unclear.” These were items with a clear hyperonym where the individual words were less typical for this hyperonym, for example “paint brush” and “file/ rasp” for TOOLS or cases where, in retrospect, the hyperonym struck us as too generic, like PERSONS, where a more fitting hyperonym would have been PROFESSIONS. Figure 6 visualizes how the gain is distributed across these different types of items. While words from items with conventional hyperonyms are evenly distributed across negative and positive gain, more words from the ad hoc hyperonyms contribute to the overall recall benefit for contrastive intonation (i.e., positive gain). Even more strikingly, all words from the unclear categories contribute to the overall recall benefit. These observations suggest that the beneficial value of focus intonation for forming an alternative set is particularly high for items that do not normally co-occur. Focus indicates the presence of alternatives, which causes a listener to scan the linguistic environment for possible alternatives. If the resulting set is one that already co-occurs often, like “apples, pears, plums,” the additional effect of focus for creating a salient set is not very strong. However, for items that have no or no strong previous relationship, focusing one of them causes the listener to view them as members of a set (the alternative set), thereby increasing their salience. The view that contrastive focus marking does not (or only minimally) increase recall for sets of co-hyponyms received is further supported by the overall recall performance (averaged across the two levels of the focus condition), which was highest for hyponyms of conventional hyperonyms (mean = 59.4%, SD = 14.7), followed by recall for elements from ad hoc hyperonyms (mean = 53.5%, SD = 8.7) and unclear cases (mean = 47.7%, SD = 15.5). Note that our experiment was not designed to test these questions; further research investigating the effect of focus on memory recall for different types of alternative sets will be needed to systematically address this question.

## Discussion

In a delayed-recall experiment, we presented equal numbers of male and female participants with auditory discourses in two different versions and assessed their memory for contextual alternatives as a function of the focus structure (broad focus, contrastive focus), which was conveyed prosodically (H+!H* vs. L+H* pitch accents realized on the focused item). The analyses show that recall for contextual alternatives was facilitated by contrastive focus. Interestingly, this effect was mainly driven by female participants. In what follows, we first discuss our findings with regard to the memory representation of discourse and different means of focus marking. Second, we discuss the finding that women showed a greater effect of prosodic focus marking than men in our task.

### Memory representations and means of focus marking

Previous studies (Fraundorf et al., 2010; Spalek et al., 2014) had already revealed that explicit focus marking improves memory for focus alternatives. Fraundorf et al. (2010) had shown that contrastive focus improved recognition memory for alternatives. Specifically, their participants were better able to reject claims that were true for the focused item but not for the alternative if the focused element had been produced with a contrastive pitch accent. By contrast, Spalek et al. (2014) had demonstrated that *recall* for contextual alternatives is facilitated in the presence of focus particles (e.g., “only the apples”), that is, in cases of “conventionalized” focus sensitivity (Beaver & Clark, 2008). In both of these studies, as well as in the present study, the focused element in the critical sentence was already contrastive for contextual reasons, independent of its prosodic realisation^3^: In the set-up sentence, a list of three elements is mentioned (e.g., tables, beds, shelves; see example 5). In the critical sentence, one of these three elements is repeated. Thus, a listener is bound to ask herself or himself: “And what about the other two things?” Therefore, what the present study (and those by Fraundorf et al., 2010, and Spalek et al., 2014) investigated was not an effect of focus per se but whether additional explicit marking of the focused element increased the salience of its alternatives. Arguably, prosodic focus cues signal focus less reliably since pragmatic inferences caused by a pitch accent can be cancelled – and might not be drawn by all individuals in the first place. The focus particle is an additional, non-negligible marker and as its lexical meaning requires access to focus alternatives, it makes the presence of alternatives more salient, as argued by Spalek et al. (2014). The present study’s result that prosodic focus facilitates *recall* for contextual alternatives but not the focused element complements the previous findings in two aspects. First, the results of Fraundorf et al. (2010) were replicated, but with a *recall* task that requires full recollection of the studied episode. In addition, the alternative set consisted of two elements in the study by Fraundorf and colleagues and of three elements in the present study, which poses even stronger demands on memory. Second, our results deliver empirical evidence in favor of the contrast account (e.g., Braun & Tagliapietra, 2010; Fraundorf et al., 2010) and to the disadvantage of the granularity account (cf. Sanford et al., 2006) as recall for contextual alternatives was boosted whereas memory for the focused element was not improved by focus marking in our study. This means that although a focused element may be encoded more specifically (see the review by Cutler et al., 1997; Sanford et al., 2006; Wang et al., 2011), related words (i.e., contextual alternatives) are not suppressed. In fact, focus seems to trigger a deeper semantic processing and/or enhance attention processes spreading benefit *especially* to the members of a contrast set. Note that we are specifically referring to Experiment 3 in Fraundorf and colleagues. In their first two experiments, where the authors probed recognition of the focused element, L+H* accent did improve memory for the focused element. We can only speculate why we did not obtain a memory benefit for the focused element itself in our recall study. While Fraundorf and colleagues quizzed participants on what happened in their stories (and what did not, the title of Fraundorf et al., 2013), we quizzed them on the content of the entire alternative set. Put differently, if we partition the stimuli in set-up sentence and critical sentence, we probed for the content of the set-up sentence, whereas Fraundorf and colleagues probed for the content of the critical sentence. Thus, a strategic component might have entered into processing such that participants in Fraundorf et al. oriented their attention more to the focused element whereas our participants concentrated more on the three list items.

Attentive readers may have noticed that we tested approximately three times the number of participants as in the precursor study (i.e., Spalek et al., 2014, Experiment 2). The reason for this was that we expected prosodic focus to yield smaller effect sizes compared to focus using focus particles. In addition, we tested *two* experimental groups, male and female listeners, which also made it necessary to increase the sample size. The effect size of prosodic focus on contextual alternative recall for our *gender-balanced* sample was approximately 3.5% compared to 4.5% effect size for the focus particle manipulation in Spalek et al. (2014, Experiment 2: 33 participants, 12 male). However, in a recent corrigendum to the original article, Spalek et al. report that, after correcting several coding errors, the effect in Experiment 2 (that is, the one we had based our study on) was no longer significant. Descriptively, the advantage had shrunk to 2% (while a 6% advantage was still present after corrections in their Experiment 1). Thus, the question whether focus signaled by focus particles triggers enhanced recall for discourse representations compared to focus cued by focus accenting remains undecided. Future studies may investigate this issue by using both focus types, contrasting the one manipulation against the other with the same test population.

Are there alternative explanations for the memory benefit caused by contrastive intonation? As an anonymous reviewer has pointed out to us, rather than claiming that focus improved recall for alternatives, we can only say that acoustic salience improved memory for focus alternatives, since the critical element in the third sentence was already “inherently” contrastive due to the way the stimuli were set up. This is in line with Calhoun (2009), who argues that there are constructions that are inherently contrastive (she uses the term “kontrastive”) but that the salience of the members of the alternative sets is affected by prominence. In particular, she claims that “[t]he more prominent a word than expected (on the basis of its syntactic/discourse properties), the more salient the alternative set, and therefore the more likely a contrastive reading” (p. 74), and also with the “effort code” described by Gussenhoven (2004), who argues that increased effort in production such as wider excursion of the pitch movement is often interpreted to express the “significance” of what is being said. This, in turn, is most often grammaticalized in the function of (contrastive) focus. The anonymous reviewer suggested the presence of contrastive intonation might not actually improve recall of focus alternatives. Rather, since the listener expects to hear an L+H* accent, its absence might hamper processing. Thus, rather than observing a memory benefit for alternatives with contrastive focus intonation, we might observe a memory detriment for alternatives with neutral intonation. We agree that it is notoriously difficult to determine the direction of an effect if only two conditions are compared. However, since it was the recall for alternatives (and not the recall for the focused element) that was affected by the experimental manipulation, the conclusion that intonation affects the salience of the mental representation of an alternative set and, therefore, its later recall, is still justified.

### Individual differences in memory for focus alternatives

As a secondary research question, we had set out to compare the performance of males and females in recall of focus alternatives. We have found both a main effect of sex such that women remembered significantly more alternatives than men and an intonation × sex interaction effect such that women benefited more from the prosodic cue than men did. The main effect is in line with reports of female superiority in episodic memory tasks with verbal materials outlined in the introduction (e.g., Herlitz et al., 1997; Herlitz & Rehnman, 2008; Lewin et al., 2001).

Resolving the sex × intonation interaction effect revealed that only the female group showed a significant memory gain if the focused element has been contrastively accented. In a recent replication study of the present finding in Vietnamese (Tjuka et al., 2020), the authors also found an interaction of sex × intonation, and observe that, even descriptively, the memory benefit for contrastive focus on alternative recall is only present for the female sample. However, as Fig. 5 illustrates, there are individuals among both males and females who show a large gain, individuals in both groups who do not show any gain, and individuals who show a reverse gain. Thus, it has to be kept in mind that the following discussion applies to the groups, not to an individual male or female listener.

Our result that male listeners do not make use of prosodic focus for the recall of heard discourse as much as female participants do is in line with the finding that sex differences in language processing are more marked during the later stages of processing related to semantic processing and interpretation (cf. Wang et al., 2011; Wirth et al., 2007). Given the nature of our task where participants had to listen to three-sentence stories and later answer recall questions about these stories, it is not surprising that this should be another task domain where sex differences obtain. Two reasons why males might make less use of the prosodic information can be assumed: Either they do not perceive it as clearly as females do or perceiving the prosodic information does not lead them to the same interpretation.

There is some evidence that men and women differ both in the intonational patterns they use themselves in speaking and in their processing of intonation: Haan and van Heuven (1999) describe the intonation patterns of Dutch speakers in different types of utterances. The female range on the majority of acoustic measures was wider than the male range. Similar findings are reported by Daly and Warren (2001) for New Zealand English. These authors report a greater pitch range and greater pitch dynamics for women than for men, and this effect is more pronounced in story telling than in reading aloud sentence lists. These differences in production might in part be able to explain differences in sensitivity for intonation, supporting the first assumption that the male group did not perceive prosodic focus as clearly as the female group did.

As discussed in the *Introduction*, Schirmer et al. (2002) reported that male listeners do not integrate prosodic and semantic information as quickly as female listeners do. If male listeners cannot exploit intonation to the same extent as female listeners, their discourse representations may also differ: Delayed or incomplete integration of the prosodic information might have precluded increases in salience in the mental representation of the alternatives for the male group. Therefore, the two conditions would not have seemed different to them, which explains the null effect. The assumption that the male group processed both intonation conditions in the same way pragmatically is even more likely because, logically, the prosodic realization of the focused element does not add any new information about its information structural status – contextual information alone is sufficient to interpret the focused element as contrastive (i.e., contrasting with the other two list items).

There is, however, an alternative explanation for the reduced effect on alternative recall in the male group: Recall accuracy *generally* reduced in male compared to female participants for both contextual alternatives and focused items. Thus, reduced overall recall in male participants might have resulted in less statistical power to observe a focus effect in this group (floor effect). The attempt to identify influential subgroups underlying the focus × sex interaction with an additional distribution analysis was not successful. This analysis rather indicated that continuous traits may play a role in contrastive focus processing across participant sex. Auditory working memory and sustained attention may be candidate measures to be included in future studies elaborating on our findings.

## Conclusion

Prosodic focus conveyed by intonation improved subsequent recall memory for alternatives to the focused element. This finding supports accounts like the contrast account that posit that focus signals the relevance of alternatives for the interpretation of an utterance, thereby increasing the salience of these alternatives in a listener’s mind. The memory improvement was only observed for women, not for men, in accordance with sex differences reported for memory, discourse processing, and the integration of intonational information with language interpretation.

## Figures and Tables

**Fig. 7:**
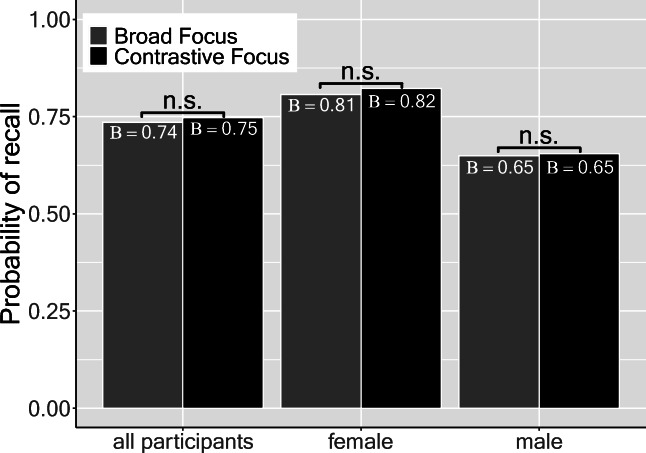
Model predictions for recall probability of the focused element. Bar charts split by focus. **Left:** Pooled results. **Middle:** Female data. **Right:** Male data

## Appendix

**Table 4:** Experimental stimuli

Stimulus number	Texts and recall cues (questions)	
	German	English translation
01	Mathias erhielt ein Paket mit Jacken^2.36^, Hosen^3.10^ und Hemden^3.01^. Er überlegte sich, was ihm am besten stand. Er hat die Hemden/**HEM****DEN** behalten. Q: Welche Kleidungsstücke waren im Paket?	Mathias received a parcel with jackets, trousers, and shirts. He wondered what would suit him best. He kept the shirts/**SHIRTS**. Q: Which clothes were there in the parcel?
02	Carsten griff in einen Korb mit Pfirsichen^0.81^, Kirschen^1.23^ und Bananen^1.18^. Er überlegte sich, worauf er Appetit hatte. Er hat die Pfirsiche/**PFIR****SICHE** herausgeholt. Q: Welches Obst lag zunächst im Korb?	Carsten reached for a basket full of peaches, cherries, and bananas. He considered what he liked. He took out the peaches/**PEACHES**. Q: Which pieces of fruit were there in the basket at first?
03	Angelika holte aus dem Supermarkt Wasser^5.28^, Cola^-0.16^ und Saft^2.10^. Sie wollte ihren Durst stillen. Sie hat das Wasser/**WA****SSER** kaltgestellt. Q: Welche Getränke wurden aus dem Supermarkt geholt?	Angelika bought water, coke, and juice at the supermarket. She wanted to quench her thirst. She cooled the water/**WATER**. Q: Which beverages were fetched from the supermarket?
04	Peter sah im Zoo Zebras^-0.52^, Löwen^2.75^ und Affen^2.34^. Er wollte sich später daran erinnern. Er hat Affen/**A****FFEN** fotografiert. Q: Welche Tiere gab es im Zoo?	Peter watched zebras, lions, and monkeys in the zoo. He wanted to remember that. He took pictures of monkeys/**MONKEYS**. Q: Which animals were there in the zoo?
05	Jens zählte in seinem Baumarkt Pinsel^1.68^, Sägen^1.03^ und Feilen^-0.08^. Er stellte fest, dass manches fehlt. Er hat Sägen/**SÄ****GEN** nachbestellt. Q: Welche Werkzeuge wurden im Baumarkt gezählt?	Jens counted brushes, saws, and files in his DIY store. He noticed that some tools are missing. He reordered saws/**SAWS**. Q: Which tools were counted in the DIY store?
06	Sarah ordnete eine Umzugskiste mit Bleistiften^1.97^, Linealen^0.48^ und Scheren^1.65^. Sie überprüfte, was nicht mehr zu gebrauchen ist. Sie hat die Bleistifte/**BLEI****STIFTE** weggeschmissen. Q: Welche Büromaterialien lagen zunächst in der Umzugskiste?	Sarah arranged a packing case full of pencils, rulers, and scissors. She checked what she did not need anymore. She threw away the pencils/**PENCILS**. Q: Which office supplies were there in the removal box at first?
07	Anja kam in ein Musikzimmer mit Geigen^1.95^, Gitarren^0.96^ und Harfen^0.57^. Sie wollte ihren Musikunterricht vorbereiten. Sie hat die Gitarren/**GI****TA****RREN** gestimmt. Q: Welche Instrumente gab es im Musikzimmer?	Anja entered a music room with violins, guitars, and harps. She wanted to prepare her music lesson. She tuned the guitars/**GUITARS**. Q: Which instruments were there in the music room?
08	Caroline betrachtete in ihrer Schatulle Ketten^3.33^, Ringe^3.27^ und Broschen^-0.03^. Sie überlegte, was zu ihrem Outfit passt. Sie hat die Ringe/**RIN****GE** herausgenommen. Q: Welche Schmuckstücke befanden sich zunächst in der Schatulle?	Caroline looked at the necklaces, rings, and brooches in her casket. She wondered what would go well with her outfit. She took out the rings/**RINGS**. Q: Which valuables were there in the casket at first?
09	Martin entdeckte im Geräteraum Reifen^1.93^, Matten^1.31^ und Seile^2.01^. Er überlegte, welche Übungen er machen wollte. Er hat Reifen/**REI****FEN** herausgeholt. Q: Welche Sportgeräte lagen im Geräteraum?	Martin discovered hoops, mats, and ropes in the gym. He decided which exercises he would like to do. He got out hoops/**HOOPS**. Q: Which pieces of sports equipment were there at the gym?
10	Doris sah im Schuppen Spaten^1.38^, Besen^1.33^ und Harken^-0.23^. Sie überlegte, was sie gebrauchen kann. Sie hat Harken/**HAR****KEN** mitgenommen. Q: Welche Gartengeräte standen im Schuppen?	Doris spotted spades, brooms, and rakes in the shed. She wondered what she would need later on. She picked up rakes/**RAKES**. Q: Which gardening tools were there in the shed?
11	Stefan sah im Waffenmuseum Dolche^1.28^, Pistolen^2.26^ und Speere^2.53^. Er war sehr interessiert. Er hat Pistolen/**PIS****TO****LEN** fotografiert. Q: Welche Waffen befanden sich im Waffenmuseum?	Stefan discovered daggers, pistols, and spears in the arms museum. He was keenly interested. He took pictures of pistols/**PISTOLS**. Q: Which weapons were there at the arms museum?
12	Michael hatte im Kulturbeutel Seife^2.04^, Shampoo^-2.10^ und Duschgel^-4.81^. Er wollte sich waschen. Er hat die Seife/**SEI****FE** ausgepackt. Q: Welche Hygieneartikel befanden sich zunächst im Kulturbeutel?	Michael had soap, shampoo, and shower gel in his toilet bag. He wanted to have a wash. He took out the soap/**SOAP**. Q: Which toiletries were there in the toilet bag at first?
13	Anna betrachtete im Möbelgeschäft Tische^4.94^, Regale^1.87^ und Betten^4.63^. Sie überlegte, was in ihre Wohnung passen könnte. Sie hat Regale/**RE****GA****LE** ausgesucht. Q: Welche Möbel gab es im Möbelgeschäft?	Anna looked at tables, shelves, and beds in the furniture shop. She considered what would look nice in her apartment. She chose shelves/**SHELVES**. Q: Which pieces of furniture were there in the furniture shop?
14	Maria fand im Spülbecken Schüsseln^2.31^, Töpfe^2.54^ und Pfannen^1.46^. Sie überlegte, was sie zum Kochen brauchte. Sie hat die Pfannen/**PFANNEN** abgewaschen. Q: Welche Küchenutensilien waren zunächst im Spülbecken?	Maria found bowls, pots, and pans in the sink. She wondered what she would need for cooking. She washed the pans/**PANS**. Q: Which cooking utensils were there in the sink at first?
15	Max suchte in seinem Kinderzimmer nach Murmeln^0.30^, Kreiseln^0.46^ und Bällen^2.75^. Er konnte nicht alles finden. Er hat die Murmeln/**MUR****MELN** verbummelt. Q: Welche Spielzeuge wurden im Kinderzimmer gesucht?	Max looked for marbles, spinning tops, and balls in his nursery. He wasn’t able to find everything. He had lost the marbles/**MARBLES**. Q: Which toys had been looked for in the nursery?
16	Janine betrachtete in der Ausstellung Statuen^2.08^, Gemälde^2.82^ und Fotografien^1.33^. Sie sollte eine Rezension schreiben. Sie hat die Gemälde/**GE****MÄL****DE** erwähnt. Q: Welche Kunstwerke gab es in der Ausstellung?	Janine looked at statues, paintings, and photographs at the exhibition. She had to write a review. She mentioned the paintings/**PAINTINGS**. Q: Which art objects were there at the exhibition?
17	Florian testete im Elektrogeschäft Mikrowellen^-1.47^, Fritteusen^-3.42^ und Toaster^-1.76^. Er überlegte, was er noch gebrauchen kann. Er hat Toaster/**TOAS****TER** gekauft. Q: Welche Geräte standen im Elektrogeschäft zur Auswahl?	Florian tried out microwaves, chip pans, and toasters in the electrical shop. He wondered what he would need. He bought toasters/**TOASTERS**. Q: Which electric devices were there in the electrical shop?
18	Katharina stand vor einem Gemüseregal mit Paprikas^1.03^, Gurken^1.49^ und Karotten^-0.08^. Sie überlegte, was sie noch zu Hause hat. Sie hat Gurken/**GUR****KEN** mitgenommen. Q: Welches Gemüse gab es im Gemüseregal?	Katharina looked at bell peppers, cucumbers, and carrots in the vegetables section. She considered what she still had at home. She bought cucumbers/**CUCUMBERS**. Q: Which vegetables were there in the vegetables section?
19	Felix begutachtete in seinem Garten Erbsen^1.60^, Bohnen^1.84^ und Zwiebeln^2.35^. Er pflegte den Garten regelmäßig. Er hat die Erbsen/**ERB****SEN** gegossen. Q: Welches Gemüse gab es im Garten?	Felix examined peas, beans, and onions in his garden. He took care of the garden regularly. He watered the peas/**PEAS**. Q: Which vegetables were there in the garden?
20	Mark öffnete eine Dose mit Bonbons^1.09^, Keksen^0.17^ und Lutschern^-2.17^. Er verspürte Lust auf Süßes. Er hat Kekse/**KEK****SE** gegessen. Q: Welche Süßigkeiten waren in der Dose?	Mark opened a jar with candies, cookies, and lollipops in it. He wanted to eat something sweet. He ate cookies/**COOKIES**. Q: Which sweets were there in the jar?
21	Susanne hatte auf ihrem Blumenbeet Rosen^3.02^, Lilien^0.73^ und Nelken^0.95^. Sie wollte einen Strauß verschenken. Sie hat Nelken/**NEL****KEN** geschnitten. Q: Welche Pflanzen waren auf dem Blumenbeet?	Susanne grew roses, lilies, and carnations on her flower bed. She wanted to give someone a bouquet. She cut carnations/**CARNATIONS**. Q: Which plants were there on the flower bed?
22	Karl jagte auf der Wiese Bienen^2.46^, Fliegen^2.54^ und Mücken^1.32^. Er hatte Spaß dabei. Er hat Fliegen/**FLIE****GEN** gefangen. Q: Welche Insekten waren auf der Wiese?	Karl chased bees, flies, and mosquitos in the meadow. He had a lot of fun. He caught flies/**FLIES**. Q: Which insects were there in the meadow?
23	Isabell notierte auf ihrer Einkaufsliste Käse^2.34^, Eier^3.93^ und Milch^3.50^. Sie hatte nicht viel Zeit. Sie hat den Käse/**KÄ****SE** vergessen. Q: Welche Nahrungsmittel standen auf der Einkaufsliste?	Isabell noted cheese, eggs, and milk on her shopping list. She was in a hurry. She forgot to buy the cheese/**CHEESE**. Q: Which groceries were there at the shopping list?
24	Torsten züchtete auf seinem Bauernhof Hühner^2.48^, Ziegen^1.79^ und Kühe^2.73^. Er überlegte, was er bereits erledigt hat. Er hat die Ziegen/**ZIE****GEN** gefüttert. Q: Welche Tiere gab es auf dem Bauernhof?	Torsten bred hens, goats, and cows on his farm. He considered what he had already taken care of. He had fed the goats/**GOATS**. Q: Which animals were there on the farm?
25	Lisa suchte im Wald Füchse^2.53^, Rehe^1.10^ und Igel^0.77^. Sie war lange unterwegs. Sie hat Igel/**I****GEL** gesehen. Q: Welche Tiere wurden im Wald gesucht?	Lisa looks for foxes, deer, and hedgehogs in the woods. She had a long walk. She saw hedgehogs/**HEDGEHOGS**. Q: Which animals had been looked for in the woods?
26	Simon las im Märchenbuch von Hexen^2.05^, Prinzen^3.79^ und Drachen^1.25^. Er las gerne vor dem Einschlafen. Er hat von Hexen/**HE****XEN** geträumt. Q: Welche Märchenfiguren kamen im Märchenbuch vor?	Simon read about witches, princes, and dragons in the storybook. He liked reading before going to bed. He dreamed of witches/**WITCHES**. Q: Which fairy-tale characters were there in the storybook?
27	Sebastian holte aus dem Wäschekorb Socken^1.19^, Pullover^1.36^ und Kleider^3.87^. Er schaute nach, was besonders dreckig war. Er hat die Pullover/**PUL****LO****VER** eingeweicht. Q: Welche Kleidungsstücke lagen zunächst im Wäschekorb?	Sebastian took out socks, sweaters, and dresses from the laundry basket. He checked what was most dirty. He soaked the sweaters/**SWEATERS**. Q: Which clothes were there in the laundry basket at first?
28	Paula betrachtete im Schuhgeschäft Stiefel^2.72^, Sandalen^0.57^ und Turnschuhe^-0.48^. Sie überprüfte, was sie sich leisten kann. Sie hat Turnschuhe/**TURN****SCHUHE** anprobiert. Q: Welche Schuhe gab es im Schuhgeschäft?	Paula looks at boots, sandals, and sneakers at the shoe shop. She considered what she could afford. She tried on sneakers/**SNEAKERS**. Q: Which kind of shoes were there at the shoe shop?
29	Julia durchsucht eine Schublade nach Taschen^3.64^, Schals^1.27^ und Hüten^3.72^. Sie wollte aufräumen. Sie hat Taschen/**TA****SCHEN** aussortiert. Q: Welche Accessoires befanden sich in der Schublade?	Julia browsed for bags, scarves, and hats in her drawer. She wanted to tidy up. She sorted out bags/**BAGS**. Q: Which accessories were there in the drawer?
30	Daniela nahm aus dem Kühlschrank Äpfel^2.58^, Birnen^1.69^ und Pflaumen^0.89^. Sie wollte backen. Sie hat die Birnen/**BIR****NEN** abgewaschen. Q: Welches Obst war zunächst im Kühlschrank?	Daniela took out apples, pears, and plums from the fridge. She wanted to bake a cake. She rinsed the pears/**PEARS**. Q: Which fruits were there in the fridge at first?
31	Leoni pflanzte auf ihrem Balkon Krokusse^-0.46^, Dahlien^-0.49^ und Veilchen^0.88^. Sie überlegte, was sie noch tun muss. Sie hat die Veilchen/**VEIL****CHEN** gedüngt. Q: Welche Pflanzen wuchsen auf dem Balkon?	Leoni grew crocuses, dahlias, and violets on her balcony. She wondered what she still had to take care of. She gave fertilizer to the violets/**VIOLETS**. Q: Which plants grew there on the balcony?
32	Falk fand in seinem Modellbaukasten Züge^4.83^, Boote^3.41^ und Schiffe^4.48^. Er überlegte, was er am liebsten machen möchte. Er hat Züge/**ZÜ****GE** zusammengebaut. Q: Welche Fahrzeuge waren im Modellbaukasten?	Falk discovered trains, boats, and ships in his model kit. He wondered what he would like to do. He assembled trains/**TRAINS**. Q: Which vehicles were there in the model kit?
33	Cornelia fand auf dem Sperrmüll Sofas^2.38^, Stühle^3.92^ und Truhen^1.28^. Sie wollte ihre Wohnung umgestalten. Sie hat Stühle/**STÜH****LE** mitgenommen. Q: Welche Möbel waren auf dem Sperrmüll?	Cornelia spotted couches, chairs, and chests in the bulky waste. She wanted to rearrange her apartment. She took chairs/**CHAIRS** with her. Q: Which pieces of furniture were there at the bulky waste?
34	Erik betrachtete im Musikgeschäft Pauken^1.08^, Flöten^1.49^ und Cellos^0.01^. Er war auf der Suche nach einem neuen Hobby. Er hat Cellos/**CE****LLOS** ausprobiert. Q: Welche Instrumente wurden im Musikgeschäft angeschaut?	Erik looked at kettledrums, flutes, and cellos in the music store. He was looking for a new hobby. He tried out cellos**/CELLOS**. Q: Which musical instruments had been looked at in the music store?
35	Petra legte auf ihren Schreibtisch Füller^-1.07^, Blöcke^3.08^ und Locher^-1.76^. Sie musste etwas vorbereiten. Sie hat Füller/**FÜL****LER** benutzt. Q: Welche Büromaterialien waren auf dem Schreibtisch?	Petra put fountain pens, blocks, and hole punches on her desk. She had to prepare something. She used fountain pens/**FOUNTAIN PENS**. Q: Which office supplies were there at the desk?
36	Robert suchte in seiner Werkstatt Zangen^1.30^, Hämmer^2.39^ und Schrauben^1.45^. Er suchte eine Weile. Er hat die Zangen/**ZAN****GEN** gefunden. Q: Welche Werkzeuge wurden in der Werkstatt gesucht?	Robert searched for pliers, hammers, and screws in his garage. He searched for a while. He found the pliers/**PLIERS**. Q: Which tools had been searched for in the garage?
37	Tamara lagerte in ihrem Tresor Rubine^0.31^, Perlen^2.29^ und Saphire^-0.15^. Sie benötigte Geld. Sie hat die Perlen/**PER****LEN** verkauft. Q: Welche Wertgegenstände waren zunächst im Tresor?	Tamara had rubies, pearls, and sapphires in her vault. She needed some money. She sold the pearls/**PEARLS**. Q: Which valuables were there in the vault at first?
38	Klaus traf auf der Baustelle Maurer^1.91^, Maler^3.95^ und Schlosser^1.20^. Er wollte die Arbeit begutachten. Er hat sich mit Schlossern/**SCHLOS****SERN** unterhalten. Q: Welche Arbeiter waren auf der Baustelle?	Klaus met bricklayers, painters, and locksmiths at the construction site. He wanted to examine the work. He talked to locksmiths/**LOCKSMITHS**. Q: Which workers were there on the construction site?
39	Franziska suchte im Badezimmer nach Bürsten^1.43^, Schwämmen^1.34^ und Lappen^1.70^. Sie wollte putzen. Sie hat die Bürsten/**BÜR****STEN** gefunden. Q: Nach welchen Putzutensilien wurde im Bad gesucht?	Franziska looked for brushes, sponges, and rags in the bathroom. She wanted to clean up. She found the brushes/**BRUSHES**. Q: Which cleaning equipment had been searched for in the bathroom?
40	Norman sah im Biologiebuch Herzen^5.10^, Mägen^2.90^ und Nieren^1.79^. Er sollte Zeichnungen anfertigen. Er hat Nieren/**NIE****REN** abgezeichnet. Q: Welche Organe waren in Biologiebuch?	Norman saw hearts, stomachs, and kidneys in the biology book. He had to make drawings. He copied the kidneys/**KIDNEYS**. Q: Which organs were there in the biology book?
41	Saskia traf auf dem Wochenmarkt Bäcker^1.52^, Gärtner^1.89^ und Bauern^4.41^. Sie wollte selbst einen Stand aufmachen. Sie hat sich mit den Bauern/**BAU****ERN** abgesprochen. Q: Welche Personen waren auf dem Wochenmarkt?	Saskia met bakers, gardeners, and farmers at the market. She wanted to have her own market stall. She talked to the farmers/**FARMERS**. Q: Which persons were there at the market?
42	Ole arbeitet im Theater mit Tänzern^1.55^, Sängern^2.65^ und Künstlern^4.30^. Er plante eine neue Aufführung. Er hat Tänzer/**TÄN****ZER** engagiert. Q: Welche Personen waren im Theater?	Ole works together with dancers, singers, and artists at the theatre. He was planning a new show. He hired dancers/**DANCERS**. Q: Which persons were there at the theater?
43	Dominik traf bei der Weltmeisterschaft Ringer^-0.06^, Läufer^1.61^ und Schwimmer^1.03^. Er wollte eine Reportage drehen. Er hat die Schwimmer/**SCHWIM****MER** interviewt. Q: Welche Sportler waren bei der Weltmeisterschaft?	Dominik met wrestlers, runners, and swimmers at the World Championship. He wanted to do a report. He interviewed the swimmers/**SWIMMERS**. Q: Which sportspersons were there at the World Championship?
44	Susanne benötigte für ihr Auto Reifen^1.93^, Bremsen^2.01^ und Felgen^0.20^. Sie musste durch den TÜV kommen. Sie hat die Bremsen/**BREM****SEN** erneuert. Q: Welche Ersatzteile wurden für das Auto benötigt?	Susanne needed tires, brakes, and wheels for her car. She had to pass the MOT test. She renewed the brakes/**BRAKES**. Q: Which spare parts were needed for the car?
45	Maik suchte auf der Landkarte China^4.25^, Peru^1.69^ und Indien^3.72^. Er wollte sehen, wo er schon gewesen ist. Er hat sich an Indien/**IN****DIEN** erinnert. Q: Welche Länder wurden auf der Landkarte gesucht?	Maik searched for China, Peru, and India on the map. He wanted to see where he had already been. He remembered India/**INDIA**. Q: Which countries had been searched for on the map?

Superscript numbers indicate frequencies (occurrences per million, transformed to natural log-scale). Bold text indicates accented words, and the accented syllable (where applicable) is underlined.

**Table 5:** Coding scheme

**Hyponyms**		
*Target*	*Response*	*Coding decision*
Block/Blöcke	Schreibblock	correct
Tasche/Taschen	Handtasche	correct
**Synonyms**		
*Target*	*Response*	*Coding decision*
Duschgel	Duschbad	correct’^#^’
Gemälde	Bild	correct
Gemälde	Malerei	correct’^#^’
Harke/Harken	Rechen	correct
Hut/Hüte	Mützen	correct
Huhn/Hühner	Hennen	correct
Jacke/Jacken	Jackett	correct
Lappen	Tücher	correct
Läufer	Sprinter	correct’^#^’
Lutscher	Lolly	correct’^#^’
Kuh/Kühe	Rind	correct
Sandale/Sandalen	Sandalette	correct
Schüssel/Schüsseln	Schale	correct
Schiff/Schiffe	Dampfer	correct
Spaten	Schaufel	correct
Statue/Statuen	Skulpturen	correct
Schüssel/Schüsseln	Teller	correct
Schal/Schals	Tuch/Tücher	correct
Turnschuh/Turnschuhe	Sportschuhe	correct’^#^’
Zug/Züge	Eisenbahnen	correct

Table 5 lists our coding specifications. Synonyms and composite hyponyms with the form “x + target word” (e.g., “Handtasche” for “Tasche”; “hand bag” for “bag”) were coded as correct responses. An answer counted as synonym if it appeared as synonym for the target word or vice versa in a German synonym reference (Duden, 2007, 2014, 2018) with few exceptions where the Duden reference did not include an entry for the target and/or the response (marked with ‘^#^’).

**Table 6:** Fixed effect estimates (top) and variance estimates (bottom) for GLMER of recall for focused items (recall accuracy ~ focus + sex + list + (1|word) + (1 + focus | participant), n=4133, log-likelihood: -2259), Coding scheme: sum coding

Fixed effects	Coefficient (*B*)	*SE*	*|z|*	*p*
Intercept	1.05	0.14	7.44	
Focus	0.06	0.09	0.65	.513
Sex	-0.84	0.21	4.07	< .001
List	-0.51	0.21	2.45	.014
Focus * Sex	-0.07	0.18	0.40	.686
**Random effects**	Variance			
Participant (Intercept)	0.86			
Random slope: Focus	0.21			
Word (Intercept)	0.40			

## Footnotes

1 The authors report three accounts, but we discuss only two of them here.

2 Target word frequencies were transformed to natural log-scale to normalize their distribution. Since some frequencies could not be obtained, model comparison via a likelihood-ratio test was not possible since the models were not fitted to the same data set. Instead, we investigated the summary of the model including frequency and found that the effect of frequency on recall was not significant (B = 0.08, se = 0.08, z = 0.96, p = .33).

3 We thank an anonymous reviewer for pointing this out to us.
